# Live nanoscopic to mesoscopic topography reconstruction with an optical microscope for chemical and biological samples

**DOI:** 10.1371/journal.pone.0207881

**Published:** 2018-12-12

**Authors:** Olivier Theodoly, Nicolas Garcia-Seyda, Fréderic Bedu, Xuan Luo, Sylvain Gabriele, Tâm Mignot, Joanna Giermanska, Jean-Paul Chapel, Mélinda Métivier, Marie-Pierre Valignat

**Affiliations:** 1 LAI, Aix Marseille Univ, CNRS, INSERM, Marseille, France; 2 CINAM, Aix Marseille Univ, CNRS, Marseille, France; 3 Univ Mons, Res Inst Biosci, Interfaces & Complex Fluids Lab, Mechanobiol & Soft Matter, Mons, Belgium; 4 LCB, Aix Marseille Univ, CNRS, Marseille, France; 5 CRPP, Univ Bordeaux, CNRS, Pessac, France; 6 Nanolane, Le Mans, France; LAAS-CNRS, FRANCE

## Abstract

Macroscopic properties of physical and biological processes like friction, wetting, and adhesion or cell migration are controlled by interfacial properties at the nanoscopic scale. In an attempt to bridge simultaneously investigations at different scales, we demonstrate here how optical microscopy in Wet-Surface Ellipsometric Enhanced Contrast (Wet-SEEC) mode offers imaging and measurement of thin films at solid/liquid interfaces in the range 1–500 nm with lateral optical resolution. A live, label-free and noninvasive methodology integrated with microfluidic devices allowed here characterization of polymers and proteins patterns together with corresponding phenotypes of living cells.

## Introduction

Physicists have constantly developed novel techniques to investigate the microscopic and nanoscopic world in immersed conditions. Optical microscopy, which came historically first, has benefited from constant innovative evolutions and is still widely used by biologists and physicists. Besides restrictions on lateral resolution due to diffraction, optical microscopy detection is principally limited by the lack of contrast of specimen, which is often colorless and translucent, for instance living cells or thin films of organic material. Since detectors in optical microscopy (the human eye or CCD sensors) are sensitive to intensity and color differences, several strategies have been developed to enhance the relative contrast of images by decreasing the background intensity. In fluorescence microscopy, background is lowered by separating illumination and emission light with a spectral emission filter, and in total internal reflection fluorescence[1] (TIRF) or confocal[2] fluorescence microscopy background, noise is further lowered by restricting the spatial excitation and detection area. Following a different strategy, differential interference contrast mode (DIC)[3],[4] and phase contrast mode (PC)[5] enhance image contrast by transforming phase changes induced by a transparent specimen into intensity differences. Nevertheless, all these techniques suffer from important limitations. Fluorescence microscopy is sensitive to photo-bleaching and necessitates labelling, which is not always possible and often biases biochemical properties of the specimen. As for DIC and PC modes, they are well adapted to visualize microscopic details but they are inadequate to detect thin transparent objects. We previously introduced a novel optical microscopy method that requires no labelling and allows detection of samples with nanometric thickness on solid transparent substrates immersed in liquid[6]. This method, called Surface Enhanced Ellipsometric Contrast in liquid or Wet-SEEC, yields observation of micrometric objects together with surface properties at the scale of a few nanometers. Here, we further show that detection, imaging and quantification capabilities of Wet-SEEC for thin films can be extended to thicknesses between one to a few hundreds of nanometers, linking the gap between the microscopic and nanometric world on a standard microscopy set-up. The methodology for wide range quantification uses home-made calibration samples composed of nano-steps fabricated by electron lithography. The calibration process compares images of nano-steps at three different wavelengths with an absolute modelling of the optical signal for convergent light reflecting in multilayered specimen. Coupling of Wet-SEEC technique and microfluidic devices allowed us here to image and measure with an optical microscope the adsorption kinetics of protein monolayer, the automated sequential deposition of polyelectrolyte multilayers, and the phenotype of living cells adapting to or modifying their microenvironment at the nanoscopic scale.

## Materials and methods

### Wet-SEEC imaging

**Fig 1:**
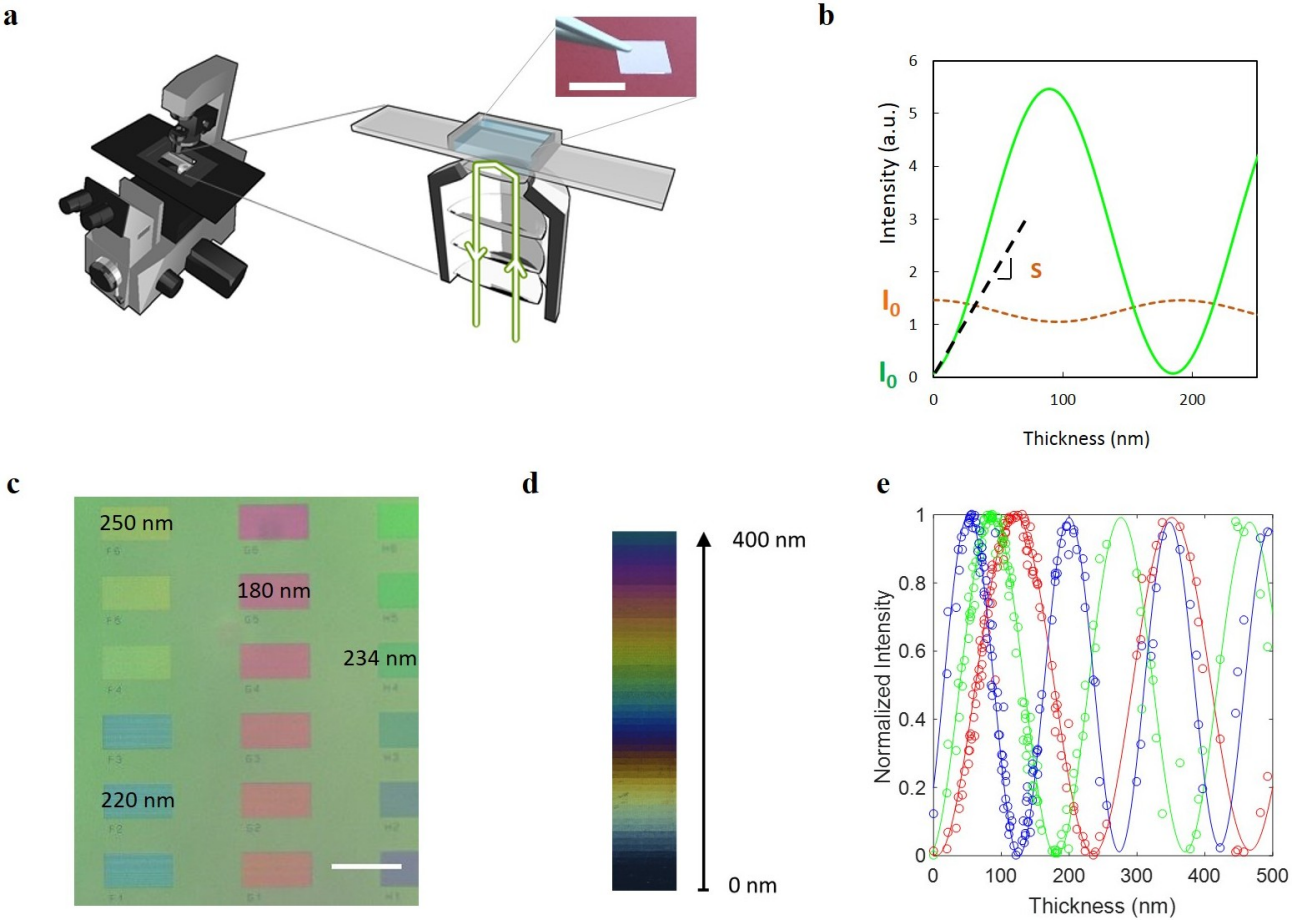
Wet-SEEC set-up, principle, calibration and measurement. **(a)** Schematics of a Wet-SEEC set up: observations on an immersed wet-surf (insert) are made in reflection mode with a standard inverted microscope. Scale bar 1 cm. **(b)** Theoretical reflected intensity for a layer of optical index *n*_*Layer*_ = 1.5 and wavelength λ = 546 nm versus the layer thickness on a glass slide (dashed orange line) and on a wet-surf (green line). **(c)** Widefield Wet-SEEC image of a resin steps calibration sample. The sample is illuminated with a white source coupled to a triple-wavelength bandpass filter and the image is taken with a x20 objective. Scale bar 50 μm. **(d)** Color scale composed of cropped Wet-SEEC images of many resin steps with different thicknesses. Images were taken with a x63 oil objective and recorded for three wavelengths. Each individual image is a merge of images recorded with three wavelengths λ = 432 nm, λ = 546 nm, and *λ* = 645 nm. Thickness scale is indicated by black arrow. **(e)** Modeling of the optical signal. Experimental (open circles) and theoretical (solid line) intensities of thin resin films of different thickness on a wet-surf after normalization by the experimental and theoretical *I*_*min*_ and *I*_*max*_ values (Eq 2). Calculations correspond to layers of refractive index 1.49 at wavelengths λ = 432 nm (blue), *λ* = 546 nm (green), and *λ* = 645 nm (red) without any adjusting parameters.

Wet-surfs are composed of a microscope coverslip bearing a layer of MgF2 of thickness 52 nm and a layer of SiO_2_ of thickness 40 nm. They were optimized to achieve anti-reflective conditions in reflection microscopy assuming a conical illumination. Microscopy images were acquired either with a Zeiss axiovert 200 inverted microscope (Carl Zeiss, Jena, Germany) equipped either with objective 63X oil immersion objective (Zeiss Neofluar 63/1.25 antiflex) for imaging/reconstruction experiments, a 50X (Zeiss NC Epi plan 50/0.75) for adsorption kinetics experiments and a 20X (Zeiss Plan-Apochromat 20x0.8) for widefield imaging. The microscope is equipped with a motrized aperture diaphragm to control the illumination cone projected into the objective. We used a CCD camera (Coolsnap HQ2; Photometrics) with chromatic filters for all multi-wavelengths Wet-SEEC measurements except for colored S1 and S2 Movies and Fig 1c for which a color camera (3 CCD DSP Sony) was used. Samples were illuminated with a fiber-coupled illuminator source (PE-300, CoolLED Ltd.,UK) with narrow filters (Chroma, USA) piloted by a filter wheel changer (Lambda 10–3, Sutter Instrument, Novato, CA, USA) or triple-wavelength bandpass filters (brightline triple-band 464/542/639 nm, Semrock, NY, USA).

### Calibration sample fabrication process

A wet-surf was first silanized with hexamethyldisilazane (Sigma-aldrich) in vapor phase, a positive tone resist of polymethyl methacrylate (PMMA 950K ARP 679.04, Allresist, Germany) was then spin-coated at 2000 rpm and baked at 170°C for 10 min to obtained a 400 nm resist thick film. A conductive layer (SX PC 9005.2 Allresist, Germany) was then spin-coated at 4000 rpm and baked at 90°C for 2 min to obtain a 40 nm thick film. E-beam lithography was performed using a Pioneer system from RAITH company (Germany) with 5kV exposures at nominal dose D_n_ = 5 μC.cm^-2^. Samples were then developed in methyl–isobutyl ketone (MIBK) mixed with isopropyl alcohol (IPA) at a ratio of 1:3 for 55 s and rinsed in IPA for 55 s. A dose variation from D_f_ = 0.742 x D_n_ to D_f_ = 7x D_n_, was performed in order to create several step thickness[7]. The thickness of each step was measured by contact profilomety (Dektak XT, Germany).

### Coupling with microfluidics and automation

Microfluidics channels for experiments with vesicle, polyelectrolyte and cell experiments were straight channels of dimensions 50 μm x 100 μm x 4 mm. The devices were fabricated using standard soft lithography procedures[8]. A positive mold was created by photolithography with SU-8 2050 negative resins (Microchem, Newton, MA, USA) on a silicon wafer (Siltronix, Archamps, France). A replica was made with polydimethylsiloxane, PDMS (Sylgard 184 Silicone Elastomer Kit, Dow Corning, Midland, USA). Ports to plug inlet and outlet tubings were punched with a gauge needle in the PDMS replica. The devices were finalized by sealing the PDMS piece on a wet-surf via O_2_-plasma activation (Harrick plasma, Ithaca, NY, USA) of both surfaces. The layer by layer polyelectrolyte deposition process was automatized with a Labview code piloting sequential injections of polyelectrolyte and rinsing solutions with 4 syringe pumps (RAZEL R100-E, St Albans, VT, USA), and a 6 way valves (nonapeak, Scivex, Oak Harbour, WA, USA).

### Protein adsorption

Wet-SEEC and quartz crystal microbalance (QCM) set up (QCM-D, Q-Sense E4 instrument, AB, Sweden) were used to probe protein adsorption at room temperature onto wet- surfs or silica-coated quartz crystal sensors. For QCM set up, the sensors were cleaned in a 1M SDS ultrasonic bath, rinsed with deionized water and UVO_3_ treated (UV/Ozone ProCleaner, BioForce Nanosciences) for 20 min before silanization in aminopropyltriethoxysilane (APTES) vapor for 1h and finally annealed under vacuum at 95°C for 30 min. The sequential adsorption of protein A (PA) (10 μg.mL^-1^), BSA (1 mg.mL^-1^) and IgG (100 μg.mL^-1^) was monitored by the decrease in the resonance frequency *fn* and variation of dissipation *D*_*n*_ for each odd overtones of the piezoelectric quartz crystal. In the case of thin and homogenous layers rigidly attached to the sensor surface, the quantitative relationship between the frequency change and the adsorbed areal mass increment *Δm* can be derived with the help of the Sauerbrey equation:

$$\frac{\Delta f_{n}}{n}=-\frac{2f_{0}^{2}}{\sqrt{\rho_{q}\mu_{q}}}\Delta m.$$ (1)

Where *f*_0_ is the resonant frequency of the crystal (5 MHz); *ρ*_*q*_ the quartz density (2.648 g.cm^-3^), *μ*_*q*_ the shear modulus (2.947 x 1011 g.cm^-1^s^-2^) and *n* the frequency overtone number. The various solutions were delivered with the help of a peristaltic pump at a flow rate of 0.1 mL.min^-1^ for 3 mn to flush entirely the cell with each new protein solutions. PBS solution was used to rinse the cell between protein adsorptions. For Wet-SEEC, the wet-surf was UVO_3_ treated (UV/Ozone ProCleaner, BioForce Nanosciences) for 20 min before silanization in APTES vapor for 1h and finally annealed under vacuum at 95°C for 30 min. The sequential injection of PA (10 μg.mL^-1^), BSA (1 mg.mL^-1^) and IgG (100 μg.mL^-1^) was delivered at a flow rate of 0.4 mL.min^-1^ for 1min followed by a 9 min of resting time (no more injection).

### Surface treatments

The stripes of octadecyltrichlorosilane (OTS) were printed by soft lithography[8]. The wet-surf was activated in a bath of a freshly prepared “Pirhana solution” (Sulfuric acid 98% and hydrogen peroxide 50% at a volume ratio 2:1) for 5 min, thoroughly rinsed with ultra-pure water (Millipore), and dried with ultra-pure Nitrogen. The surface of a PDMS stamp was dipped for 10 s in an OTS solution in toluene at 1 wt %, then wiped out from excess solution in a flow of pure nitrogen. This was then applied to a cleaned wet-surf for 30 s. The samples were finally sonicated in 2 sequential chloroform baths. Stripes of proteins IgG and ICAM-1-Fc (R&D Systems, Minneapolis, MN) were printed on wet-surf by the light-induced molecular adsorption (LIMA) technique[9]. Wet-surfs were first silanized with APTES in vapor phase then pegylated with PEG-SVA (M_w_ = 5000 Da, Interchim, France) at concentration 0.23 mg.mL^-1^ for 1h at room temperature. Printing zones were then activated using UV (λ = 375 nm) and activator 4-benzoylbenzyl-trimethylammonium chloride (custom synthesis by Sigma-Aldrich outsourced to SinoChem, China) using the digital micromirror device (DMD) illumination set-up, and finally exposed to sequential adsorptions of Protein A (50 μg.mL^-1^, 1h at 37 °C), BSA (4%wt, 15 min at 37 °C) and antibody IgG (50 μg.mL^-1^, 1h at 37 °C) or ICAM-1 (10 μg.mL^-1^, 1h at 37 °C) separated by rinsing sequences of PBS. The layer-by-layer (LbL) polyelectrolyte depositions were made on an initial graft layer of poly(ethyleneimine), PEI (*M*_*w*_ = 750,000, ref 181978, Sigma), and poly(sodium-4-styrene sulfonate, PSS (*M*_*w*_ = 70,000, ref 243051, sigma) and poly(allylamine), PAH (*M*_*w*_ = 65,000, ref 479144, Sigma) layers were subsequently added. Polymer solutions were prepared at 0.5% wt with 1 M of NaCl and injected. A rinsing solution of NaCl at 1M is injected between each polyelectrolyte deposition.

### Cell preparation

Whole blood from healthy adult donors was obtained from the “Établissement Français du Sang”. Peripheral blood mononuclear cells (PBMCs) were recovered from the interface of a Ficoll gradient (Eurobio, Evry, France). T cells were isolated from PBMCs with Pan T cell isolation kit (Miltenyi Biotec, Bergisch Gladbach, Germany), then were stimulated for 48 h with anti-CD3/anti-CD28 Dynabeads (Gibco by Thermo Fischer Scientific, Waltham, MA) according to the manufacturer’s instructions. T lymphocytes were subsequently cultivated in Roswell Park Memorial Institute Medium (RPMI) 1640 supplemented with 25 mM GlutaMax (Gibco, Gibco by Thermo Fischer Scientific, Waltham, MA, USA), 10% fetal calf serum (FCS; Gibco by Thermo Fischer Scientific, Waltham, MA, USA) at 37°C, 5% CO_2_ in the presence of IL-2 (50 ng.mL^-1^; Miltenyi Biotec, Bergisch Gladbach, Germany) and used 6 to 10 days after stimulation. At the time of use, the cells were >99% positive for pan-T lymphocyte marker CD3 and assessed for activation and proliferation with CD25, CD45RO, CD45RA and CD69 markers as judged by flow cytometry.

### Ethics statement

Human subjects: Blood from healthy volunteers was obtained through a formalized agreement with French Blood Agency (Etablissement Français du Sang, agreement n° 2017–7222). Blood was obtained by the agency after informed consent of the donors, in accordance with the Declaration of Helsinki. All experiments were approved by the INSERM Institutional Review Board and ethics committee.

## Results and discussion

### Calibration samples allow quantitative Wet-SEEC from nanoscopic to mesoscopic scale

The Wet-SEEC method uses a conventional inverted microscope and special substrates, called wet-surfs, that present like standard glass cover slips (Fig 1a). Observations are made by interference reflection microscopy (IRM), an optical label-free technique that is usually used to reveal nanometric thin films between a settled specimen and a glass microscope slide[10]. The detection of thin films by IRM is efficient when the refractive index of the film is lower than the one of the upper medium, e.g. beads, vesicle or living cell separated from substrate by a thin film of solvent. In contrast, the detection is poor when the film index is higher than the one of the upper medium, e.g. dense organic/inorganic films deposited on a substrate in solution (S1 Fig). These strong limitations are overcome here by the use of wet-surfs whose optical properties are optimized to achieve antireflective conditions in reflection microscopy. Wet-surfs are composed of a glass cover slip coated with multiple dielectric layers allowing antireflective conditions[11,12] with a top layer of Si0_2_ allowing all types of surface treatment procedures applicable to glass. Fig 1b compares the theoretical intensities reflected by a wet-surf and by a glass substrate versus the thickness of a deposited thin layer of refractive index *n*_*Layer*_ = 1.5. Calculation is performed for an observation in water using monochromatic light (wavelength *λ* = 546 nm) and reduced illumination numerical aperture (*INA* = 0.7). Reflection intensity on bare substrates, *I*_*0*_, is ~20 times lower on a wet-surf than on glass, which is decisive for contrast enhancement. Moreover, the slope of the reflected intensity versus film thickness at origin, *S*, is steep on a wet-surf and null on glass, which insures a sharply higher sensitivity on wet-surfs to the presence of a thin film with nanometer thickness. Furthermore, the amplitude of the intensity oscillations versus film thickness is one order of magnitude larger on a wet-surf than on glass. Therefore, Wet-SEEC images are also highly sensitive to nanometric thickness variations in mesoscopic films of thickness ranging between tens and hundreds of nanometers. Altogether, this technique provides a unique tool to link measurement and imaging of samples at nano- and meso-scopic scales simultaneously. However, like all optical interferometric methods, e.g. ellipsometry or optical waveguide light mode spectroscopy (OWLS), Wet-SEEC also faces the problem of periodicity of the experimental signal versus sample thickness. For a given wavelength, thickness is only determined with a periodicity of the order of λ/4. To solve the degeneracy of the measurements, we therefore acquired images at several wavelengths[13,14]. Additionally, to achieve absolute measurement, we developed a calibration procedure based on home-built reference samples. These reference samples consist of PMMA thin steps of different thickness that were prepared by electron beam lithography on a wet-surf (Fig 1c-left) and measured by AFM and contact profilometry. A combination of images from steps of various thickness allowed us to build an experimental scale linking tint and thickness for Wet-SEEC images in the range 0-400nm for resin steps (Fig 1d). The theoretical reflected intensities of PMMA steps in water were calculated with our optical model of Wet-SEEC[15] using the same refractive index *n* = 1.49 for wavelengths λ = 432, 546, 645 nm, and numerical aperture *INA = 0*.*7*. For direct comparison, experimental and theoretical data must however follow an appropriate normalization procedure that removes unknown experimental parameters such as the influence of the intensity level of the lighting or the response of the camera. Theoretical intensity is normalized using the first minimum, *I*_*min*_, and first maximum, *I*_*max*_, of theoretical intensity versus film thickness for each wavelength, as given by the following equation:

$$I_{N}=\frac{I-I_{\text{min}}}{I_{\text{max}}-I_{\text{min}}}.$$ (2)

Similarly, experimental intensity is normalized using PMMA calibration steps. One can check on Fig 1e that normalized intensity values of the theoretical and experimental cases match well in the entire 0–500 nm range. One can also check that a set of experimental signals at the chosen three wavelengths is unique for a given step height value. The theoretical curves of Fig 1e can therefore be used to convert a triple-wavelength Wet-SEEC image from a matrix of intensities data into a matrix of topography data with a resolution around the nanometer (S2 Fig). In all experiments, images of the calibration sample and of the sample of interest must be taken under the same optical conditions (objective, wavelength, INA…) and further flattened using a background image of a bare wet-surf. The presence of an extra reflection from a ceiling of microfluidic channel can be taken into account following the procedure described in [14].

### Calibrated Wet-SEEC allow quantitative imaging of small molecules, nanoparticles and proteins monolayers

**Fig 2:**
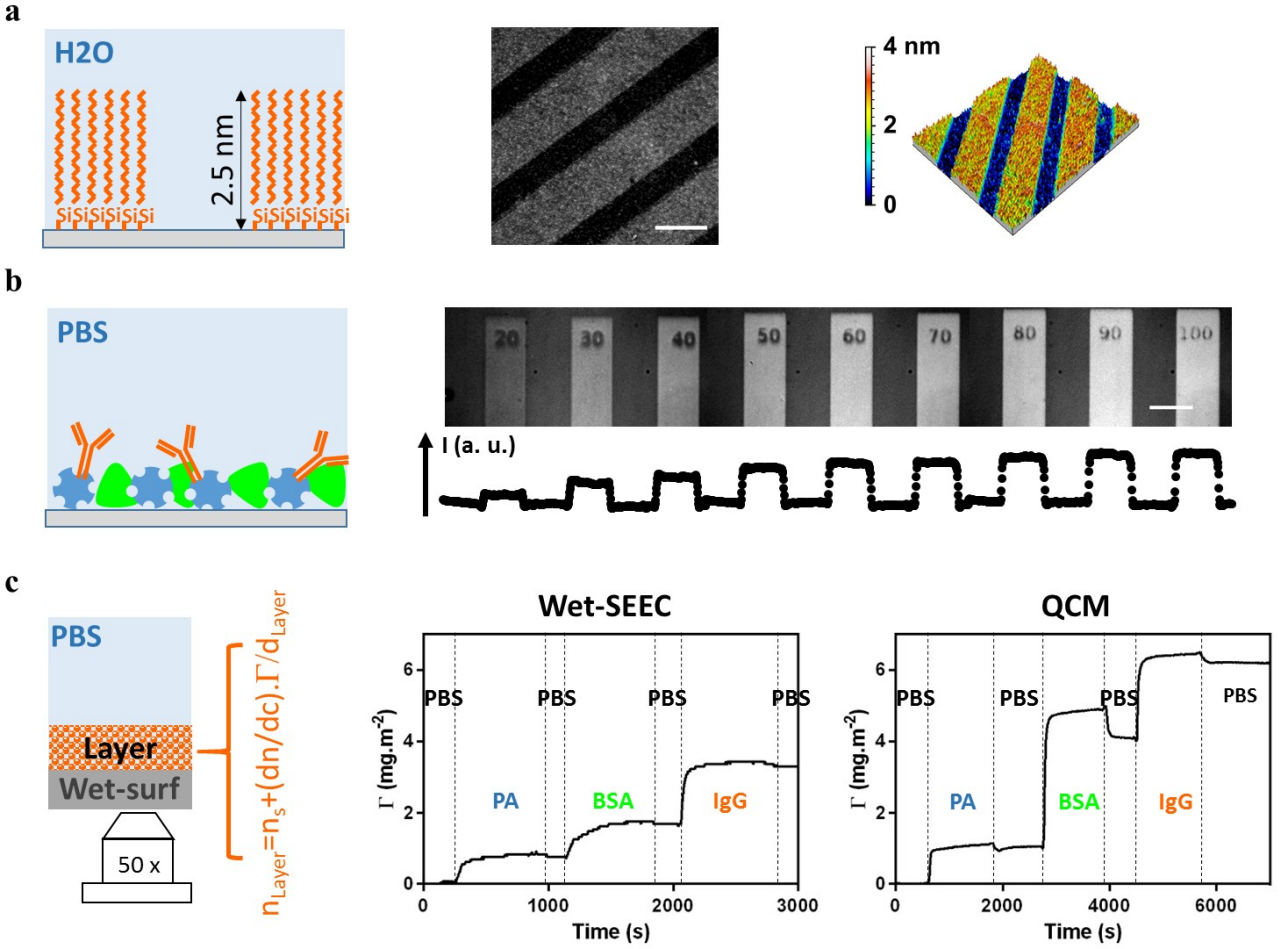
Nanoscopic measurement and imaging of dense and hydrated molecular layers. **(a)** Analysis of dense layers: Schematic of an OTS layer grafted on a wet-surf (left). Raw Wet-SEEC image of OTS microcontact-printed stripes at *λ* = 546 nm and INA = 0.7 and magnification x63 (center). 3D sample topography reconstruction from Wet-SEEC image, the OTS layer is measured at 2.1 ±0.5 nm (right). Scale bar 50 μm **(b)** Analysis of hydrated layers: Schematic of proteins adsorption layer composed of a first layer of protein A (PA) printed by the LIMA technique[9] and further adsorption of Bovine Serum Albumin (BSA) and IgG antibody (left). Wet-SEEC image at *λ* = 546 nm and INA = 0.7 and magnification x63 of protein layers obtained for different doses of printing activation (right-top). Wet-SEEC intensity profile reveals an increase of protein adsorbed amounts versus the printing activation dose (right-bottom). Scale bar 50 μm **(c)** Adsorption kinetic measurement: Schematic of the optical model for a hydrated film as a homogeneous layer of thickness and refractive index *d*_*Layer*_ and *n*_*Layer*_ (left). Adsorption kinetics measurement, adsorbed amount *Γ* vs. time, for sequential injections of PA, BSA and IgG proteins and rinsing with phosphate-buffered saline (PBS), as measured by Wet-SEEC (center) and Quartz microbalance (Right) techniques.

The performance of calibrated Wet-SEEC to characterize nanoscopic surfaces treatments is first highlighted with grafted molecular monolayers of OTS patterned on wet-surfs using micro-stamps (Fig 2a). Remarkably, OTS patterns appear clearly on raw Wet-SEEC images, and quantitative analysis yields a layer thickness of 2.1 ± 0.5 nm that compares well with the previous measurements of stamped OTS layers^10^. A 3D topography of the molecular pattern can further be reconstructed. These unmatched capabilities of Wet-SEEC to image and measure nanoscopic films immersed in water with an optical microscope apply not only to organic but also to inorganic material, as exemplified with monolayer of Cerium oxide CeO_2_ nanoparticles[16] (S3 Fig). In the domain of molecular films, the case of protein monolayers is of critical interest. Proteins at interfaces govern indeed most functions and fates of living cells versus their microenvironment, so that the control or detection of protein adsorption is crucial in numerous applied issues related to biomolecular or biomedical analysis. To test the performances of Wet-SEEC with protein layers, we printed protein patterns on wet-surfs using the optical molecular printing technique LIMA[9]. Stripes of protein A (PA) with different surface concentrations were patterned and then incubated with an IgG antibody. Image of the samples (Fig 2b) reveals clearly the stripes of proteins layers and the stripes contrast increases with the printing dose. The sensitivity of Wet-SEEC thus allows the detection of protein layers in the absence of fluorescence labelling, which is decisive, because labeling often jeopardizes the functional properties of the proteins of interest. Interestingly, the Wet-SEEC technique can also be used to determine protein adsorption kinetics, provided that imaging during the adsorption process is performed in an appropriate flow cell that allow controlled transport of adsorbates and rinsing solutions. We performed sequential adsorptions of PA, bovine serum albumin (BSA) and antibody IgG on an APTES treated wet-surf (Fig 2c). As is the case for any optical surface characterization technique, the quantitative analysis for Wet-SEEC appears less straightforward for proteins layers than for dense hydrophobic layers (e.g. silane, lipid, polystyrene). In the latter case, the refractive index of the layer corresponds to the index of pure adsorbate in such manner that the only unknown is the layer thickness. In contrast, an immersed protein layer is much more complex because it is bicomponent due to hydration and it presents a density profile versus the distance from the substrate. Following simplified models used in other optical methods (surface plasmon resonance (SPR), ellipsometry, optical reflectometry, OWLS), we assume a step-like profile for the adsorbed layer, which limits the unknown parameter to layer thickness and average refractive index of the layer. The average adsorbed amount, *Γ*, is then related to layer thickness, *d*_*Layer*_, and refractive index, *n*_*Layer*_, of the layer via:

$$n_{Layer}=n_{s}+\frac{\left(\frac{dn}{dc}\right)\Gamma }{\;d_{Layer}}.$$ (3)

where the refractive index of the pure PBS solution is *n*_*s*_ = 1.33, and the refractive index increment of a protein solution in PBS versus protein concentration *C* is $(\frac{dn}{dc})=0.185\;mL.g^{-1}$ [17]. One can check that the signal is hardly dependent on different combinations of thickness and refractive index *d*_*L*_ and *n*_*L*_ corresponding to a given average adsorbed amount *Γ*[18,19] (S4 Fig). Wet-SEEC therefore yields an average adsorbed amount of protein that can be directly compared to QCM data, for which frequency shift *vs* adsorbed amount was taken as 1 Hz corresponding to 0.1767 mg.mol^-1^ [20]. Fig 2c shows that measurements by Wet-SEEC on APTES treated wet-surfs compare well with measurements by QCM on APTES treated quartz surfaces. The adsorbed amounts match remarkably between the two techniques for PA and IgG, whereas BSA is detected in lesser amount in Wet-SEEC than in QCM. This difference may be attributed to the specific structure of BSA layers. QCM data present a net divergence of harmonics for BSA layers (S5 Fig) that reveals viscoelasticity property of the structure and therefore a strong hydration of the layer. Quantitative measurements from QCM and optical interferometric methods are known to match well for dense layers but to a lesser extent for low density layers[21]. Altogether, our results on protein adsorption show that Wet-SEEC is a powerful technique to characterize biological surface treatments with microscopic lateral resolution and nanoscopic axial resolution This may be particularly relevant for analytical measurements of solute concentrations by detection of binding on ligand-coated surfaces (the sensitivity and the limit of the technique are given in Table A in S1 File and are further discussed in S1 File). Wet-SEEC has specific assets like the small amounts of reactants required for a measurement and more importantly the submicrometric lateral optical resolution that allows combinatorial measurements on microarrays, an approach that is possible with SPR[22] but precluded in low resolution techniques such as ellipsometry, QCM.

### Polyelectrolyte multilayers exemplify in situ mesoscopic characterization with nanoscopic thickness sensitivity

**Fig 3:**
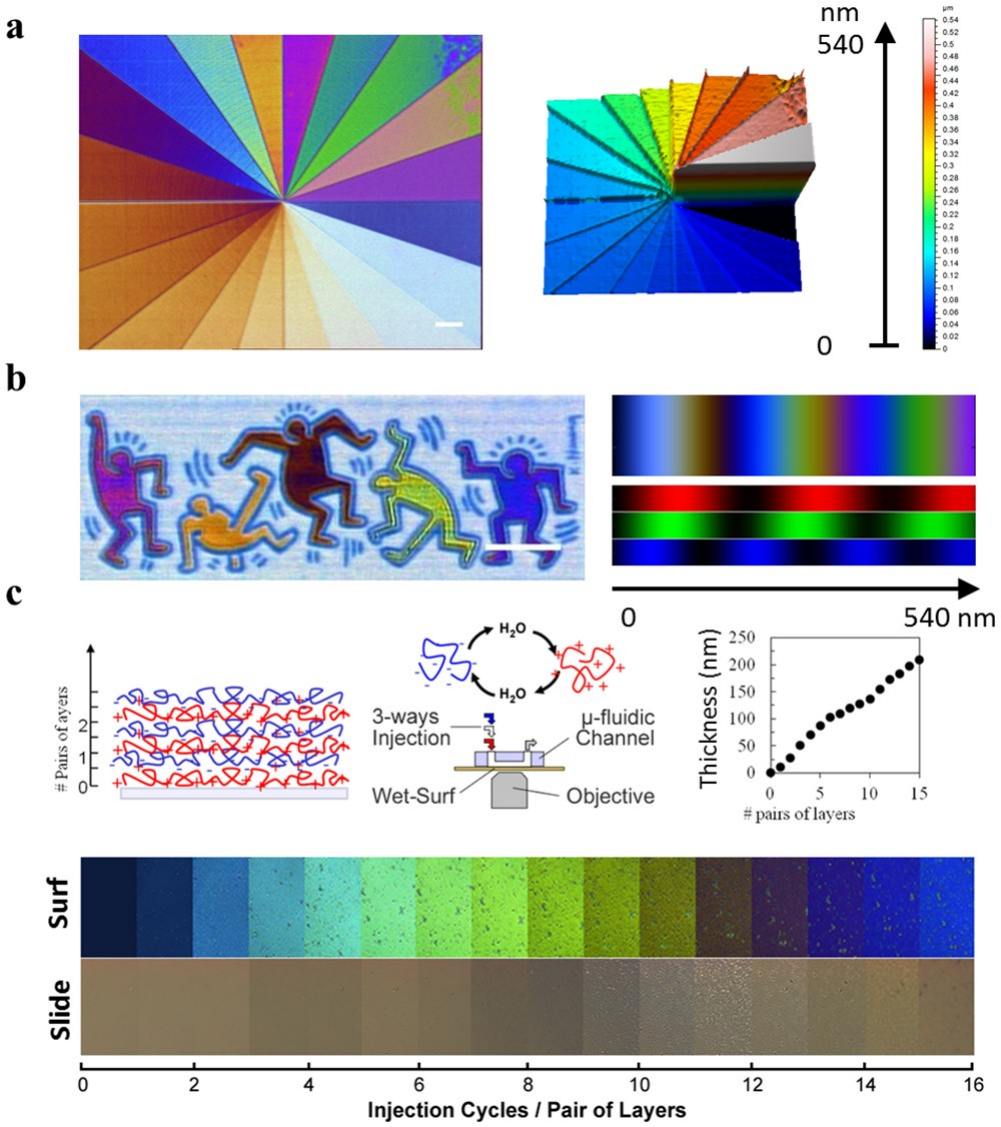
Mesoscopic measurement and imaging of dense and hydrated thin films. **(a)** Wet-SEEC image (left) and 3D Topography reconstruction based on Wet-SEEC image of a nanofabricated scale of steps 20–30 nm linking the nanoscopic and mesoscopic measurement capabilities. Scale bar 10 μm. **(b)** Raw Wet-SEEC image of a nanofabricated sample (inspired from a Keith Haring painting) composed of characters of different thickness within the mesoscopic range. The tint in the Wet-SEEC image can be directly linked to a corresponding thickness using the theoretical tint scale (right). Scale bar 10 μm. **(c)** Schematics of the automatized layer by layer polyelectrolyte (LbL) deposition in a coupled microfluidics/Wet-SEEC device (Top-left). Sequence of images versus number of deposited pairs of layers of poly(allylamine), PAH, and polystyrene sulfonate, PSS, taken on a wet-surf and on a glass microslide (Bottom). Thickness of the polyelectrolyte multilayer measured by the Wet-SEEC technique versus the number of polyelectrolyte layers (Top-right). Error bars ± 3 nm. Polyelectrolyte solutions were made at 0.5%wt with 1 M of NaCl and adjusted to pH = 2 for PAH. (See also S1 Movie).

Imaging and absolute 3D topography reconstruction of calibrated Wet-SEEC in the mesoscopic scale (up to 500 nm) is then evaluated using an electron lithography nanofabricated sample. A pie-sliced drawing was used to insulate a PMMA electron sensitive film of 550 nm thickness deposited on a silanized wet-surf, each pie slice being assigned with a different insolation time. Although the entire specimen is transparent, raw Wet-SEEC imaging with combined 3 wavelength illumination reveals a multicolored pie, reflecting directly the difference in thicknesses of pie slices (Fig 3a). Inversion of Wet-SEEC image into an absolute topographic image yields a 3D representation of nanometric helicoidally staircase composed of 20 steps of thickness 20–30 nm that connects the bare substrate to 550 nm high plateau. Beyond reconstruction of mesoscopic topography, Wet-SEEC is also relevant to detect minute fluctuations in the topography of mesoscopic samples due to the large amplitude of intensity oscillations with respect to thickness. The image of a Keith Haring painting was used to structure a PMMA film of thickness 380 nm, each different color of the painting being assigned with a slightly different insolation time. Slightly different thickness appear with sharply different tints in raw Wet-SEEC imaging with combined 3 wave length illumination (Fig 3b), as exemplified by the orange and reddish characters that have thicknesses of respectively 110 and 140 nm. Theoretical tint scales on wet-surfs can therefore be used to determine thin film thickness at a glance. Such imaging capabilities are generally relevant to study nanoscopic features in samples with mesoscopic thickness, like the wrinkling of thin films under shear or heat constraints[23]. An example of 140 nm polystyrene with riddles of amplitude 50 nm and wavelength 2 μm formed after swelling by toluene vapor is presented in (S6 Fig) Wet-SEEC is clearly offering unprecedented opportunities to study immersed films from the nanoscopic to the mesoscopic range and is also particularly relevant to follow the dynamics of structural changes in mesoscopic films in situ. This ability is used here to study systems of layer by layer (LbL) polyelectrolytes depositions, an established powerful technology to engineer innovative surface treatments with targeted physico-chemical and biological properties^18,19^. Successive expositions of a substrate to solutions of positively and negatively charged polymers leads to LbL depositions of polymer layers on top of each other (Fig 3c). LbL formation is however an out of equilibrium process that is highly dependent on processing history, and a precise characterization of LbL formation in situ is highly valuable for processing control or optimization. Here, LbLs were engineered using a microfluidic device by sequential automated injections of polymers (polyallylamine, PAH, and polystyrene sulfonate, PSS) and rinsing solutions[24]. Microfluidic processing allows precise flow rates, as well as injection and incubation durations, which is essential to insure the reproducibility of out-of-equilibrium assemblies. Changes in intensity and tints of images versus the number of depositions of polymer layers is sharply pronounced on wet-surf, whereas it is barely detectable on glass. Considering homogeneous layers with a step like profile and a refractive index of 1.49, one can deduce a linear increase of the film thickness with the number of layers up to 200 nm, the average thickness of each layer being around 7 nm[25]. Hence, these results demonstrate that Wet-SEEC is a unique innovative tool to characterize mesoscopic thin films with nanoscopic resolution. Live imaging is also particularly helpful to reveal important structural changes versus environmental changes. For instance, LbL multilayers were found to present nodulations and eventually delamination when submitted to shear stress in microfluidic devices which reveal nonhomogeneous cohesion with the material (S1 Movie).

### Calibrated Wet-SEEC links living cells phenotypes with surface nanoscopic features

**Fig 4:**
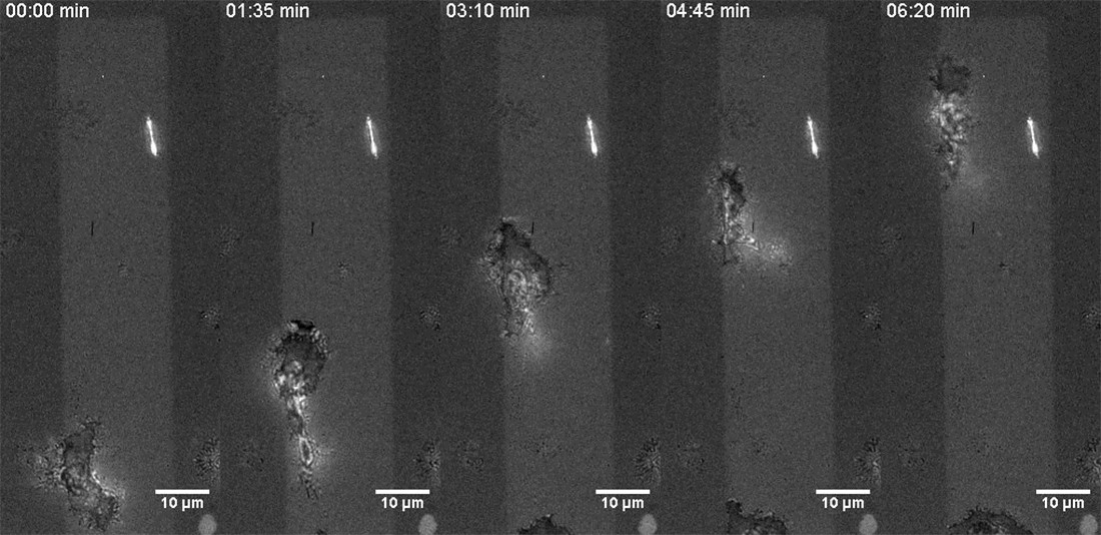
Living cells and nanoscale environment. Wet-SEEC reveals the selective adhesion and migration of human effector primary T cells on ICAM-1 adhesion molecules stripes printed by the LIMA technique and appearing in bright in the images. Non-patterned surface appears in light grey and adhesion zone of cells in black. Scale bar 10 μm (See also S2 Movie).

Novel tools are highly needed to investigate the links between living cells and their nanoscopic environment. The fate of cells and tissues is strongly influenced by the nature and distribution of adhesion molecules or haptotactic signals anchored on supporting substrates. Conversely, cells can themselves extrude and deposit nanoscopic layers of material on a substrate, e.g. to generate tissue matrix (skin, bone), to promote their own adhesive environment or to deliver guiding signals for partner cells. Combination of Wet-SEEC with other modes of microscopy opens unique opportunities to perform correlative analysis between the phenotypes of living organisms and nanoscopic modulations of their microenvironment without intrusive fluorescence labelling. To support this, wet-surfs were first patterned with 20 μm wide stripes of adhesion proteins ICAM-1 separated by 20 μm wide stripes of anti-fouling protein BSA, and then incubated with T lymphocytes expressing integrins LFA-1 molecules (that specifically binds ICAM-1). Whereas bright field transmission microscopy can only yield cell position and shape without any insight into the molecular pattering of the substrate, Wet-SEEC mode images reveals proteins patterns and the contact zones between cells and substrates (Fig 4). Intimate adhesion between cells and substrate (that correspond to dark zones below cells in Wet-SEEC images) appears exclusively on adhesive patterns of molecules ICAM-1 (that appears as brighter zones in Wet-SEEC). These data demonstrate the preferential orientation of crawling cells in the direction of the printed stripes, thus showing a direct link of a cell phenotype driven by nanoscopic molecular modulation of its environment. Such experiments are typically appropriate to decipher the guiding or activating properties of cells by chemokines and adhesion molecules. Conversely, modifications of a substrate by cells deposing a molecular layer can be directly monitored by Wet-SEEC. We previously imaged the bacteria *Myxococcus Xanthus* (MX) gliding on chitosan treated wet-surf[6]. The nanoscopic deposition of mucilage (slime) in the wake of MX cells was directly revealed by Wet-SEEC without labelling. Absence of staining is sometimes crucial to avoid any interference between labelling agent and proteins functions. In the case of MX, native slime formed preferential migrating routes for bacteria, whereas stained slime was a repellant for bacterial adhesion and migration. Label-free Wet-SEEC analyses are therefore an effective and efficient tool to further investigate how cellular secretion contributes to their adhesion, friction or communication properties.

## Conclusions

Calibrated Wet-SEEC provides an unparalleled optical method to image and quantify samples from nanoscopic to microscopic scales with minimal perturbations. It is particularly well suited for label-free, quantitative and noninvasive studies of living samples interactions with substrates features of nanoscopic thickness. Coupled to microfluidics and multiple microscopy modes, it opens multiple perspectives for the control of surface functionalization and the fundamental study of phenomena occurring at the solid-liquid interface in various scientific and technological fields of physics, chemistry and biology.

## Supporting information

S1 Fig IRM nanoscopic detection is adapted to detection of specimen height and SEEC-Wet to detection of specimen thickness. **(a)** Schematics describing the refractive index distribution of a specimen with refractive index 1.48 (green) either “suspended” (left) or “deposited” (right) on a glass substrate (grey) and immersed in aqueous solution (blue). **(b) Left—**Contrast calculated for IRM signal versus height of suspended specimen (dark blue) and thickness of deposited specimen (brown). **Middle—**IRM image of a suspended lipid vesicle. Scale bar 10 μm. -**Right**- IRM image of a deposited lipid film. Scale bar 10 μm. **(c) Left—**Contrast calculated for Wet-SEEC signal versus height of suspended specimen (dark blue) and thickness of deposited specimen (brown). **Middle–**Wet-SEEC image of a suspended lipid vesicle. Scale bar 10 μm. -**Right**- Wet-SEEC image of a deposited lipid film. Scale bar 10 μm. Supporting Methods.
(TIF)

S2 FigThickness determination and precision in Wet-SEEC.**a-** Simulation of thickness determination with normalized intensities I_r_, I_g_, I_b_ corresponding to a resin thin film of 70 nm at the 3 wavelengths of respectively *λ* = 432 nm, 546 nm, and 645 nm. The curve represents the difference *ΔI* between this triplet of values and all the triplets of values corresponding to films of thickness *h* comprised between 0 and 600 nm, following the formula $\Delta I(h)=\sqrt{{\left(I_{r}-I_{r}(h)\right)}^{2}+{\left(I_{g}-I_{g}(h)\right)}^{2}+{\left(I_{b}-I_{b}(h)\right)}^{2}}$. There is a single minimum at 0 corresponding to thickness *h =* 70 nm, which exemplifies how measurement using 3 wavelengths can solve the degeneracy issue of measurements using a single wavelength (where equivalent solutions occur every quarter wavelength). **b-** Simulation of thickness error measurement versus film thickness in the range 0 to 600 nm. We consider 5% of error in experimental normalized intensities measured for each wavelength *λ* = 432 nm, 546 nm, and 645 nm, and the thickness error corresponds to the difference between the thicknesses obtained with the exact intensities and the intensities shifted by 5%. Resolution remains within a few nanometers in the whole range from 0 to 600 nm. **c-** Sensitivity or relative change of intensity for a thickness increase of 1 nm versus the thickness of a layer of refractive index 1.4.(TIF)

S3 Fig Thin films of 10 nm diameter CeO_2_ nanoparticles. Wet-SEEC images of a thin film adsorbed from a 0.1%wt CeO_2_ nanoparticles solution with 0.1 M NaN_3_ at pH 1.5 and then rinsed with nitric acid (HNO_3_) solution at pH = 1 and deionized water. Picture is taken at the position of the contact line of the droplet used for adsorption, allowing to observe the neat frontier between the thin 10 nm dense CeO_2_ film (brighter) and the substrate (darker). The substrate is a wet-surf coated with a hexamethyldisilazane layer of thickness around 1 nm.
(TIF)

S4 Fig Wet-SEEC measurement of hydrated layers allow determination of adsorbed amount. Calculated Wet-SEEC intensities with respect to the product of the sample thickness times the difference in refractive index between the layer and the solvent, which is proportional to the adsorbed amount. The calculation is reported for refractive indexes of 1.4, 1.38 1.36 and 1.34, which corresponds to a gradual increase in the hydration state of the layer.
(TIF)

S5 Fig QCM sensistivity to layer viscoelasticity. Raw QCM data for sequential adsorption of PA, BSA and IgG proteins alternated by rinsing with PBS on a silanized QCM sensor. The divergence of the different overtones after BSA adsorption reveals the presence of a viscoelastic layer. In contrast, Wet-SEEC is in principle sensitive to the dry adsorbed amount and not to hydration or viscoelastic properties of the adsorbed layer.
(TIF)

S6 Fig Observation of thickness modulations in a mesoscopic wrinkled polystyrene film. **(a)** Wet-SEEC image sequence of a spin-coated 140 nm polystyrene film (2500 RPM, 1 minute) from a 2% wt. toluene solution, irradiated by UV/O_3_ for 10 min, then exposed to toluene vapor. Swelling occurs via defects in the layers and eventually evolves into wrinkles within 1 hour. Scale bar 5 μm. **(b)** After one hour in a saturated toluene atmosphere the thin PS film displays highly contrasted wrinkles at large planar scales. Scale bar 10 μm.
(TIF)

S1 File Supporting material. Table A. Lower and upper limits of validity for the quantitative measurement of adsorbed amount with hydrated layers.
(DOCX)

S1 Movie Growth and delamination of a polyelectrolyte layer by layer (LbL) film in a microfluidics channel. The movie shows Wet-SEEC Image sequence during automatized LbL polyelectrolyte deposition in a coupled microfluidics/Wet-Surf device, using combined wavelengths *λ* = 432 nm, *λ* = 546 nm, and 645 nm. Each novel sequential image corresponds to the addition of a pair of deposited layers of poly(allylamine), PAH, and polystyrene sulfonate, PSS. Polyelectrolyte solutions we made at 0.5%wt with 1 M of NaCl and adjusted to *pH* = 2 for PAH (see Material and methods). Adsorption and rinsing were made at flow rates of 0.1 mL.min^-1^ for 10 min. Imaging reveals several features of film structure and sensitivity to flow: The film thickness is different in the center and in the edges of the channel, where solute transport and hydrodynamic shear stress conditions are different. Thick films present also the formation of macroscopic nodulations that eventually lead to complete delamination of a fraction of film. The substrate after delamination is still covered by a multilayer which reveals nonhomogeneous cohesion in the LbL films.
(AVI)

S2 Movie Live imaging without staining of a eukaryotic migration influenced by its nanoscopic environment. Wet-SEEC reveals the selective adhesion and migration of human effector primary T cells on patterned ICAM-1 adhesion molecules stripes. Stripes were printed using the LIMA technique and appear bright in the images. Non-patterned, and therefore antifouling stripes, are covered by Polyethylene glycol (PEG) coating and appear in light grey in the images. The adhesion zone of cells appears in black.
(AVI)
