# Supplementary figures and images for: Live nanoscopic to mesoscopic topography reconstruction with an optical microscope for chemical and biological samples
Source: PLoS One. 2018 Dec 12;13(12):e0207881. doi: 10.1371/journal.pone.0207881 (PMC6291091; doi:10.1371/journal.pone.0207881)

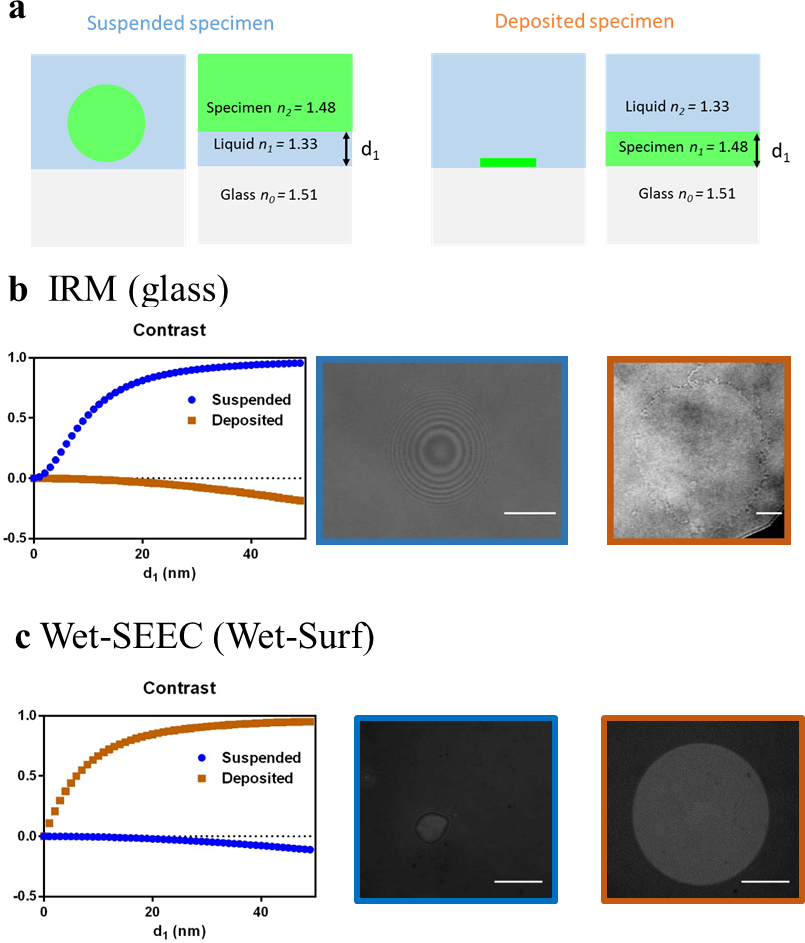

Supplement: S1 Fig — (a) Schematics describing the refractive index distribution of a specimen with refractive index 1.48 (green) either “suspended” (left) or “deposited” (right) on a glass substrate (grey) and immersed in aqueous solution (blue). (b) Left—Contrast calculated for IRM signal versus height of suspended specimen (dark blue) and thickness of deposited specimen (brown). Middle—IRM image of a suspended lipid vesicle. Scale bar 10 μm. -Right- IRM image of a deposited lipid film. Scale bar 10 μm. (c) Left—Contrast calculated for Wet-SEEC signal versus height of suspended specimen (dark blue) and thickness of deposited specimen (brown). Middle–Wet-SEEC image of a suspended lipid vesicle. Scale bar 10 μm. -Right- Wet-SEEC image of a deposited lipid film. Scale bar 10 μm. Supporting Methods. (TIF) [file pone.0207881.s001.tif]

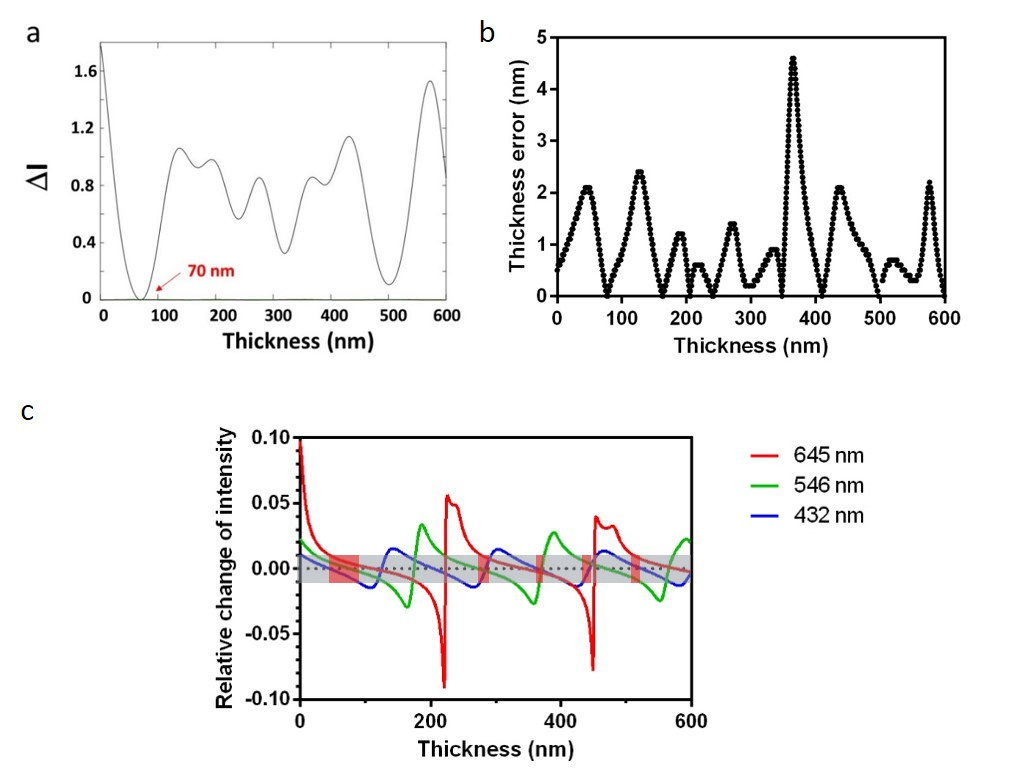

Supplement: S2 Fig — a- Simulation of thickness determination with normalized intensities Ir, Ig, Ib corresponding to a resin thin film of 70 nm at the 3 wavelengths of respectively λ = 432 nm, 546 nm, and 645 nm. The curve represents the difference ΔI between this triplet of values and all the triplets of values corresponding to films of thickness h comprised between 0 and 600 nm, following the formula ΔI(h)=(Ir-Ir(h))2+(Ig-Ig(h))2+(Ib-Ib(h))2. There is a single minimum at 0 corresponding to thickness h = 70 nm, which exemplifies how measurement using 3 wavelengths can solve the degeneracy issue of measurements using a single wavelength (where equivalent solutions occur every quarter wavelength). b- Simulation of thickness error measurement versus film thickness in the range 0 to 600 nm. We consider 5% of error in experimental normalized intensities measured for each wavelength λ = 432 nm, 546 nm, and 645 nm, and the thickness error corresponds to the difference between the thicknesses obtained with the exact intensities and the intensities shifted by 5%. Resolution remains within a few nanometers in the whole range from 0 to 600 nm. c- Sensitivity or relative change of intensity for a thickness increase of 1 nm versus the thickness of a layer of refractive index 1.4. (TIF) [file pone.0207881.s002.tif]

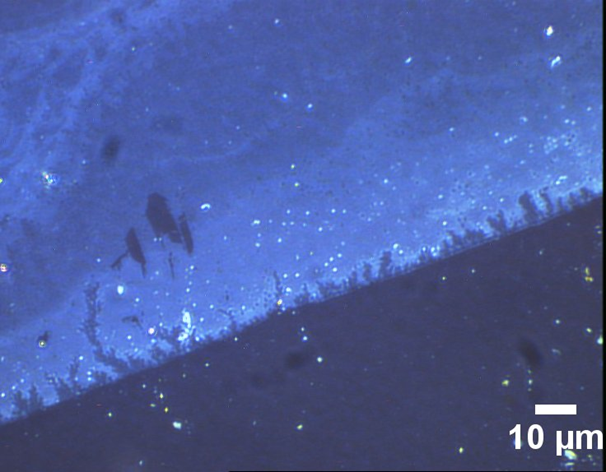

Supplement: S3 Fig — Wet-SEEC images of a thin film adsorbed from a 0.1%wt CeO2 nanoparticles solution with 0.1 M NaN3 at pH 1.5 and then rinsed with nitric acid (HNO3) solution at pH = 1 and deionized water. Picture is taken at the position of the contact line of the droplet used for adsorption, allowing to observe the neat frontier between the thin 10 nm dense CeO2 film (brighter) and the substrate (darker). The substrate is a wet-surf coated with a hexamethyldisilazane layer of thickness around 1 nm. (TIF) [file pone.0207881.s003.tif]

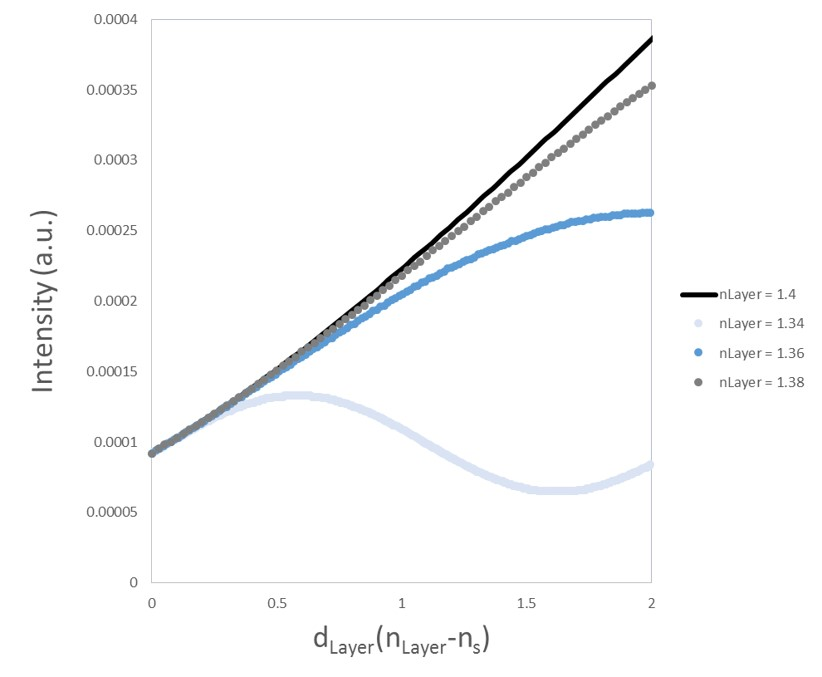

Supplement: S4 Fig — Calculated Wet-SEEC intensities with respect to the product of the sample thickness times the difference in refractive index between the layer and the solvent, which is proportional to the adsorbed amount. The calculation is reported for refractive indexes of 1.4, 1.38 1.36 and 1.34, which corresponds to a gradual increase in the hydration state of the layer. (TIF) [file pone.0207881.s004.tif]

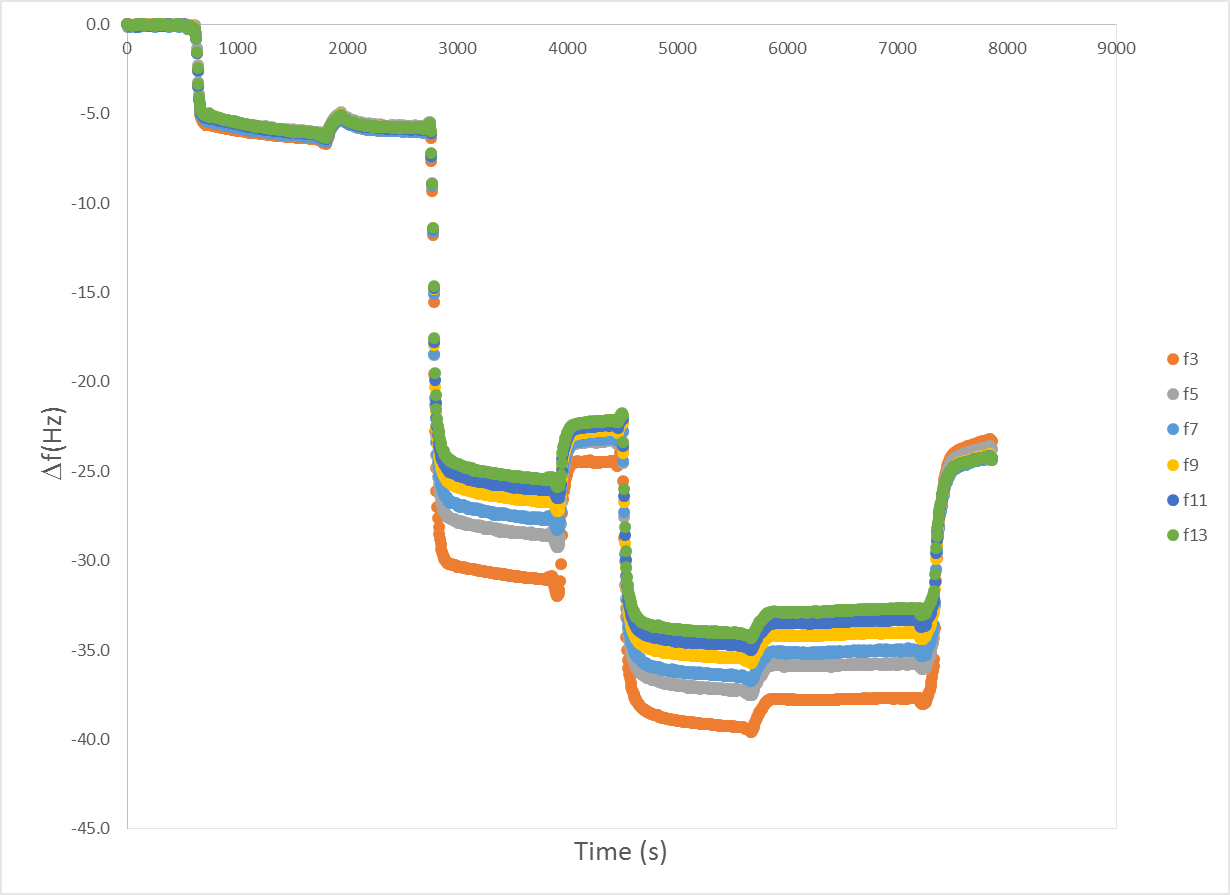

Supplement: S5 Fig — Raw QCM data for sequential adsorption of PA, BSA and IgG proteins alternated by rinsing with PBS on a silanized QCM sensor. The divergence of the different overtones after BSA adsorption reveals the presence of a viscoelastic layer. In contrast, Wet-SEEC is in principle sensitive to the dry adsorbed amount and not to hydration or viscoelastic properties of the adsorbed layer. (TIF) [file pone.0207881.s005.tif]

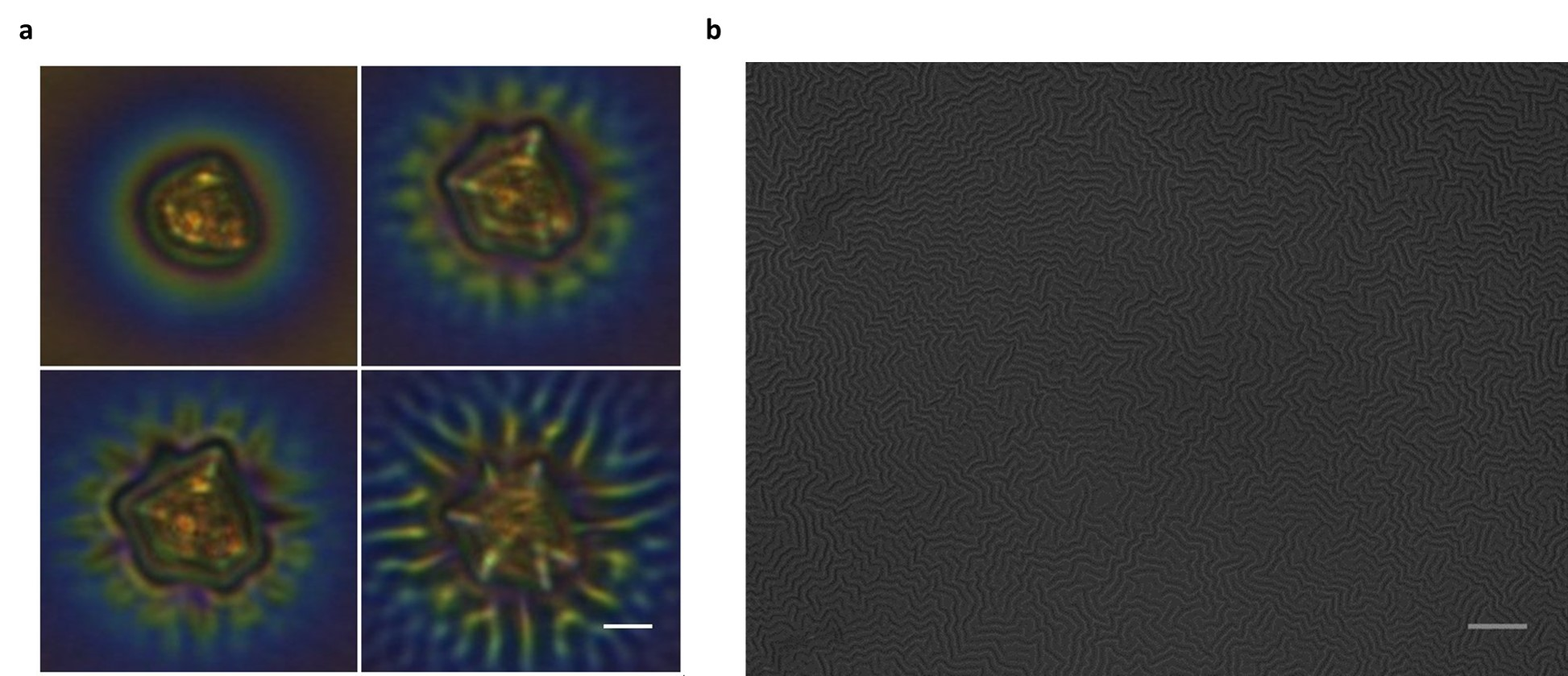

Supplement: S6 Fig — (a) Wet-SEEC image sequence of a spin-coated 140 nm polystyrene film (2500 RPM, 1 minute) from a 2% wt. toluene solution, irradiated by UV/O3 for 10 min, then exposed to toluene vapor. Swelling occurs via defects in the layers and eventually evolves into wrinkles within 1 hour. Scale bar 5 μm. (b) After one hour in a saturated toluene atmosphere the thin PS film displays highly contrasted wrinkles at large planar scales. Scale bar 10 μm. (TIF) [file pone.0207881.s006.tif]
