# Supplementary material for: Live nanoscopic to mesoscopic topography reconstruction with an optical microscope for chemical and biological samples
Source: PLoS One. 2018 Dec 12;13(12):e0207881. doi: 10.1371/journal.pone.0207881 (PMC6291091; doi:10.1371/journal.pone.0207881)
Supplement: S1 File — Table A. Lower and upper limits of validity for the quantitative measurement of adsorbed amount with hydrated layers. (DOCX) [file pone.0207881.s007.docx]

**SUPPORTING MATERIAL**

**IRM Imaging.** IRM is an optical label free technique often used to study cell adhesion or cell mobility or to measure nanometric distances between objects and a glass coverslip. In IRM, a light source is focused by a microscope objective on to the surface of a surface and a nearby object. The reflections from the substrate and the object, collected through the same objective, interfere to form a pattern that encodes the optical distance between the two surfaces. The total reflected intensity can be approximately written as , where is the phase shift between the two reflections and $r_{ij}=\frac{n_{i}-n_{j}}{n_{i}+n_{j}}$ is the reflection coefficient at the interface between media *i* and *j* (S1 Fig.**-a**). What makes IRM a powerful technique to study (for instance) cell adhesion (or suspended specimen, S1 Fig.**-a)** is that the refractive index of a living cell (*n_2_* = 1.37 to 1.48) is close to the refractive index of the glass (*n_0_* = 1.50 to 1.54). This has two implications: (i) the amplitude reflected by both interfaces are similar and (ii) the intensity in the absence of liquid layer (*d_1_* =0) is close to zero. Hence the contrast $C=\frac{I(d_{1})-I(d_{1}=0)}{I(d_{1})+I(d_{1}=0)}$ between the intensity reflected by the cell with or without a liquid layer is large and allows detection of nanometric distances between a cell and a substrate. The situation is strongly different if one wants to detect a thin layer of material on a substrate in immersed condition (Deposited specimen, S1 Fig.**-a**). Medium 2, the liquid, has a refractive index close to 1.3 while the specimen of thickness *d_1_* has an intermediate refractive index, for instance 1.48 between the refractive index of the substrate and the upper medium. This has two implications (i) the amplitude reflected by both interfaces are different and (ii) the intensity in the absence of organic layer (*d_1_* =0) is different from zero and therefore the contrast is low.

**Wet-SEEC Imaging.** In Wet-SEEC, the coverslide is bearing two layers of transparent materials. The total reflected intensity writes where and is the phase for$d_{1}=0$. The Wet-Surfs are designed such as and $r_{01}$ , the total reflection coefficient at the interface specimen / Wet-Surf (deposited specimen, S1 Fig.**-a**) is close in amplitude to that of the reflection coefficient between the deposit and the surrounding medium $r_{12}$ (antireflective layer). Since $r_{01}$and $r_{12}$are small compare to unity, the total reflected intensity can be approximatively written as

$$I(d_{1})\approx\left(\left|r_{01}\right|^{2}+\left|r_{12}\right|^{2}\right)\left(1-\frac{2\left|r_{01}\right|\left|r_{12}\right|}{\left|r_{01}\right|^{2}+\left|r_{12}\right|^{2}}+\frac{\left|r_{01}\right|\left|r_{12}\right|}{\left|r_{01}\right|^{2}+\left|r_{12}\right|^{2}}\left(\frac{4\pi n_{1}d_{1}}{\lambda}\right)^{2}\right)$$

Therefore, the intensity scales quadratically with the thickness of the deposit over a near zero background and the contrast can be made arbitrarily large by decreasing $1-\frac{2\left|r_{01}\right|\left|r_{12}\right|}{\left|r_{01}\right|^{2}+\left|r_{12}\right|^{2}}$ .

In this study, the contrast has been optimized by taking into account the geometry of the incident beam i.e. by integrating incident beam over the cone of illumination.

**IRM and Wet-SEEC modes for nanoscopic detection of specimen height and specimen thickness.** When considering a specimen in aqueous solution with a refractive larger to water, its optical detection close to glass substrate depends strongly whether this specimen is “suspended” in the vicinity of the substrate (with a thin separation film of aqueous solution) or if it is “deposited” on the substrate (S1 Fig. a). We performed here theoretical simulations of optical contrast versus height/thickness of specimen in the suspended and deposited case for a lipid vesicle specimen (n_2_=1.48) in aqueous solution (n_1_= 1.33) in the vicinity of a glass substrate (n_0_=1.51). Quantitatively S1 Fig. b shows that theoretical contrasts versus height/thickness in IRM mode are large for suspended specimen and very low for deposited films. This is confirmed by images of suspended vesicle, whose contact zone shows contrasted Newton rings, and of a deposited lipid bilayer that is barely visible. In contrast, S1 Fig. c shows that theoretical contrasts versus height/thickness in Wet-SEEC mode are low for suspended specimen and very high for deposited films. This is confirmed by images of a suspended vesicle, whose Newton rings in the contact zones are less contrasted than in IRM mode, and of a deposited lipid bilayer that displays a very high contrast.

**Thickness determination in Wet-SEEC using three wavelengths**. To determine the thickness of a deposited specimen based on three images taken at three different wavelengths, one has to find the best match between the normalized experimental intensities and the normalized theoretical intensities calculated versus specimen thickness (like the ones of Fig 1-d in the main text). As an example S2 Fig.**-a** shows a simulation of thickness determination for a resin film of 70 nm assuming a refractive index of 1.5. We consider the theoretical normalized intensities *I_r_*, *I_g_*, *I_b_* for 3 wavelengths *λ* = 432 nm, 546 nm, and 645 nm. The curve represents the difference Δ*I* between this triplet of values and all triplets of values corresponding to films of thickness *h* comprised between 0 and 600 nm, following the formula $\Delta I(h)=\sqrt{\left( I_{r}-I_{r}(h) \right)^{2}+\left( I_{g}-I_{g}(h) \right)^{2}+\left( I_{b}-I_{b}(h) \right)^{2}}$. There is a single minimum at 0 for thickness *h =* 70 nm. This exemplifies how a measurement using 3 wavelengths can solve the degeneracy issue of measurements using a single wavelength. Note that in this example, the minimum of $\Delta I$ is equal to zero because the minimization was performed with theoretical values. With experimental values, one usually finds a principal minimum with a value different but close to zero.

**Error in thickness determination and sensitivity of the Wet-SEEC signal to thickness change.**

Thickness error corresponds to the error in thickness measurements due to the experimental errors inherent to the method. We previously estimated the error in the experimental optical signal using microfluidic samples filled with a series of fluid of known optical indexes[1]. For a single measurement, the experimental signal was found lower than 10 % of the theoretical signal without any adjustment. For a signal calibrated using a series of steps, the error can be decreased below 5 %. The error in the signal is then transformed into an error in thickness through the optical model, which yields S2 Fig -b. The thickness error of the technique is in the end below 5 nm in the range 0 to 600 nm for a layer of known refractive index.

Sensitivity corresponds to the smallest thickness step that the technique allows to discriminate. The smallest detectable thickness step depends on the smallest optical contrast that can be detected, which is around 1 % for the human eye and for most standard camera without signal treatment. S2 Fig. -c shows the relative change of intensity (or contrast) for a thickness increase of 1 nm. The grey region on the figure stands for absolute contrast values below the detection threshold of 1 %. The red region highlights the region where the contrast is below 1% for all wavelengths. Sensitivity is therefore better than 1 nm for layers of thickness comprised between 0 to 600 nm except in the narrow red regions.

**Wet-SEEC measurement of hydrated layers**

The Wet-SEEC method cannot determine both the thickness and the refractive index of a hydrated layer but can provide quantitative information on the amount adsorbed. The experimental signal is indeed mainly sensitive to the amount adsorbed and much less to different combinations of refractive index and thickness corresponding to a fixed adsorbed amount. To characterize the range of thickness and refractive index where this approximation is valid, we consider a solvent of refractive index $n_{s}=1.33$ and a pure material of refractive index 1.4. We then calculated the signal as a function of $d_{Layer}\left(n_{Layer}-n_{s}\right)$ , which is proportional to adsorbed amount, for layers of refractive indices 1.38 ($dn=n_{Layer}-n_{s}$ = 0.06), 1.36 (*dn* = 0.03) and 1.34 (*dn* = 0.01), which corresponds to a gradual increase in the hydration state of the layer. We finally determined from S4 Fig. the limit where the signal differs by a maximum of 5% between a hydrated layer ($n_{Layer}<1.34$) and a dense layer ($n_{Layer}=1.34$). For highly hydrated layers (*dn* = 0.01), this limit is reached for$d_{Layer}\left(n_{Layer}-n_{s}\right)=0.36$, which corresponds to a layer of thickness of 36 nm for the hydrated layer. In other words, for the highly hydrated layers, the upper limit of validity of the adsorbed quantity determination method is 36 nm. By the same token, the upper limit is 22 nm for a layer of *dn* = 0.03 and 20 nm for a layer of *dn* = 0.05. The smallest detectable thickness can also be assessed, it is fixed by the limit of sensitivity of 1 % and is therefore 2 nm for$n_{Layer}=1.36$, 1.5 nm for $n_{Layer}=1.36$and 1 nm for$n_{Layer}=1.38$. Let us note that if the smallest detection thickness decreases with layer dilution, the minimum adsorbed amount remains constant and equal to 0.7 mg.m^-2^.

| **n_Layer_** | **1.34** | **1.36** | **1.38** |
| --- | --- | --- | --- |
| **Lower detection limit (nm)** | 2 | 1.5 | 1 |
| **Lower detection limit (mg.m^-2^)** | 0.7 | 0.7 | 0.7 |
| **Maximal layer thickness (nm)** | 36 | 22 | 20 |
| **Maximal adsorbed amount (mg.m^-2^)** | 3.5 | 7 | 10.5 |

**Table A.** Validity lower and upper limits for the quantitative measurement of hydrated layers. Pure adsorbed material has an index of 1.4 and solvent of 1.33.

1. Huerre A, Jullien M-C, Theodoly O, Valignat M-P. Absolute 3D reconstruction of thin films topography in microfluidic channels by interference reflection microscopy. Lab Chip. 2016;16: 911–916. doi:10.1039/C5LC01417D
